# Qualitative and quantitative differences in endometrial inflammatory gene expression precede the development of bovine uterine disease

**DOI:** 10.1038/s41598-020-75104-7

**Published:** 2020-10-26

**Authors:** Amy Brewer, Paul Cormican, Joseph J. Lim, Aspinas Chapwanya, Cliona O’Farrelly, Kieran G. Meade

**Affiliations:** 1 Animal and Bioscience Research Department, Teagasc, Grange, Co. Meath, Ireland; 2 School of Biochemistry and Immunology, Trinity College Dublin, Dublin 2, Ireland; 3 Department of Clinical Sciences, Ross University School of Veterinary Medicine, Basseterre, St Kitts and Nevis; 4 School of Medicine, Trinity College Dublin, Dublin 2, Ireland; 5 School of Agriculture and Food Science, University College Dublin, Dublin 2, Ireland

**Keywords:** Immunogenetics, Transcriptomics

## Abstract

The transcriptome of the endometrium early postpartum was profiled to determine if inflammatory gene expression was elevated in cows which subsequently developed uterine disease. Endometrial cytobrush samples were collected at 7 days postpartum (DPP) from 112 Holstein–Friesian dairy cows, from which 27 were retrospectively chosen for RNA-seq on the basis of disease classification [ten healthy and an additional 17 diagnosed with cytological endometritis (CYTO), or purulent vaginal discharge (PVD)] at 21 DPP. 297 genes were significantly differentially expressed between cows that remained healthy versus those that subsequently developed PVD, including *IL1A* and *IL1B* (adjusted *p* < 0.05). In contrast, only 3 genes were significantly differentially expressed in cows which subsequently developed CYTO. Accounting for the early physiological inflammatory status present in cows which do not develop disease enhanced the detection of differentially expressed genes associated with CYTO and further expression profiling in 51 additional cows showed upregulation of multiple immune genes, including *IL1A*, *IL1B* and *TNFA*. Despite the expected heterogeneity associated with natural infection, enhanced activation of the inflammatory response is likely a key contributory feature of both PVD and CYTO development. Prognostic biomarkers of uterine disease would be particularly valuable for seasonal-based dairy systems where any delay to conception undermines sustainability.

## Background

Following calving, the cow must undergo the heavily energy-dependent and parallel processes of uterine involution and the initiation of lactation. In addition, the open cervix, now exposing the previously gravid uterus must also rapidly transition from a state of immune suppression and relative quiescence to active immune-surveillance^1^. Foetal membranes are expelled, necrosis and sloughing of the caruncles occurs^2^, and the tissues of the shrinking uterus are regenerated. Although bacterial infection is inevitable after calving, it seems that the local immune system has a remarkable ability to support the development of a protective microbiome and restore homeostasis.

In a significant proportion of high performing cows, however, infection leads to the development of various forms of disease which delays uterine involution, and negatively impacts other aspects of the cow’s productive physiology. The most significant detrimental effects have been documented from the development of clinical uterine disease, which is usually diagnosed by a veterinarian after 21 days postpartum (DPP) by the presence of purulent vaginal discharge (PVD). PVD is associated with lower conception rates, increased services per conception and a longer calving interval^3,4^. The energy diverted into immune and inflammatory processes can also result in reduced production of milk and can result in premature culling^5^. Our work has recently shown that the negative effect of PVD extends to pasture-based dairy cows, and that higher milk yield is a significant risk factor for subsequent development of uterine disease^6^. Although prevalence varies according to type of cow and farming system, the impact of disease on seasonal based dairy systems, where cows are required to conceive within 85 days of calving to maintain an annual calving pattern, remains relatively unexplored. Sub-clinical disease referred to as cytological endometritis (CYTO) is diagnosed by quantification of polymorphonuclear (PMN) cells in the uterine endometrium^7,8^. By its nature, CYTO is more subtle in presentation, is more difficult to diagnose and therefore disease-associations have been less comprehensively documented, particularly in extensive grazing dairy systems. Nevertheless, evidence suggests a negative effect of subclinical disease on reproductive performance^9^. PVD and CYTO are not mutually exclusive forms of disease, and the interplay between disease presentations remains unclear. Even subtle negative effects on ovarian function^10^ can contribute to suboptimal fertility which would significantly undermine the sustainability of seasonal dairy systems.

Where infection interrupts the return to homeostasis in the postpartum cow, the inevitable response of the host immune system is inflammation, but these changes have not been well documented at the molecular level in cattle. Gene expression profiling of inflammatory genes has previously been performed in cows with endometritis at both circulating level in whole blood^11^ as well as locally in cytobrush or biopsy samples^12,13^. While some inflammation is regarded as a natural, or physiological response to bacterial influx^14^, unresolved or dysregulated inflammation is now recognised as central to the pathogenesis of endometritis^10,15^. In his review, LeBlanc proposes a model showing how either excessive or inadequate levels of endometrial inflammation can result in endometritis^16^. However the molecular networks regulating the activation and resolution of inflammation in the postpartum uterus as well as underpinning the divergent responses between cows have not been defined. High-throughput genomics approaches have identified significantly different endometrial gene expression profiles in cows with both types of uterine disease at 42–60 DPP^17^. Robust predictive gene expression signatures could signpost avenues to prevent the development of disease-associated pathology, however it is not known whether robust differences can be detected in the early postpartum period. Our earlier work showed the upregulation of both *IL1* and *IL17* at 7 DPP using RNA-seq in endometrial biopsies from cows that developed cytological endometritis (CYTO) compared to their counterparts which resolved inflammation^12^. These findings suggest that despite the fact that most cattle experience uterine inflammation after calving, differences may exist between physiological and pathological inflammation which signals the potential to identify cows at risk of developing subsequent uterine disease early postpartum.

Given their principal role in the activation and orchestration of the immune response, in this study, we aimed to characterise the 7 DPP endometrial cell transcriptomic expression profiles using RNA-seq of tissue from postpartum cows with more detailed disease phenotypes, with a view to defining early prognostic indicators of uterine health and disease. To achieve this objective, three distinct comparisons were undertaken: Comparison A—between cows which remained healthy and cows which subsequently developed cytological endometritis (CYTO), or purulent vaginal discharge (PVD) by 21 DPP; Comparison B—between healthy cows with physiological inflammation at 7 DPP and cows which developed CYTO by 21 DPP; and Comparison C—between cows with and without physiological inflammation at 7 DPP.

## Results

### Occurrence and impact of clinical and subclinical uterine disease in 112 postpartum dairy cows

This study was performed on 112 spring-calving, mixed-parity Holstein–Friesian cattle from a commercial dairy farm in Ireland. Disease diagnosis at 21 DPP revealed that 76% (n = 85) of cattle had one or more forms of uterine disease. Of these animals, 28% (n = 31) had CYTO, 18% (n = 20) had PVD only and 30% (n = 34) had both CYTO and PVD. The cows selected for this detailed immunological analysis were part of a larger study which assessed the effect of clinical disease on production and reproduction parameters in pasture-based dairy cattle^6^. PVD was shown in this study to significantly increase the calving to conception period (CCP) in all cows, but to a greater extent in cows with a high milk yield in the lactation before disease diagnosis, showing cumulative effects of physiological demand on reproduction indices.

**Figure 1:**
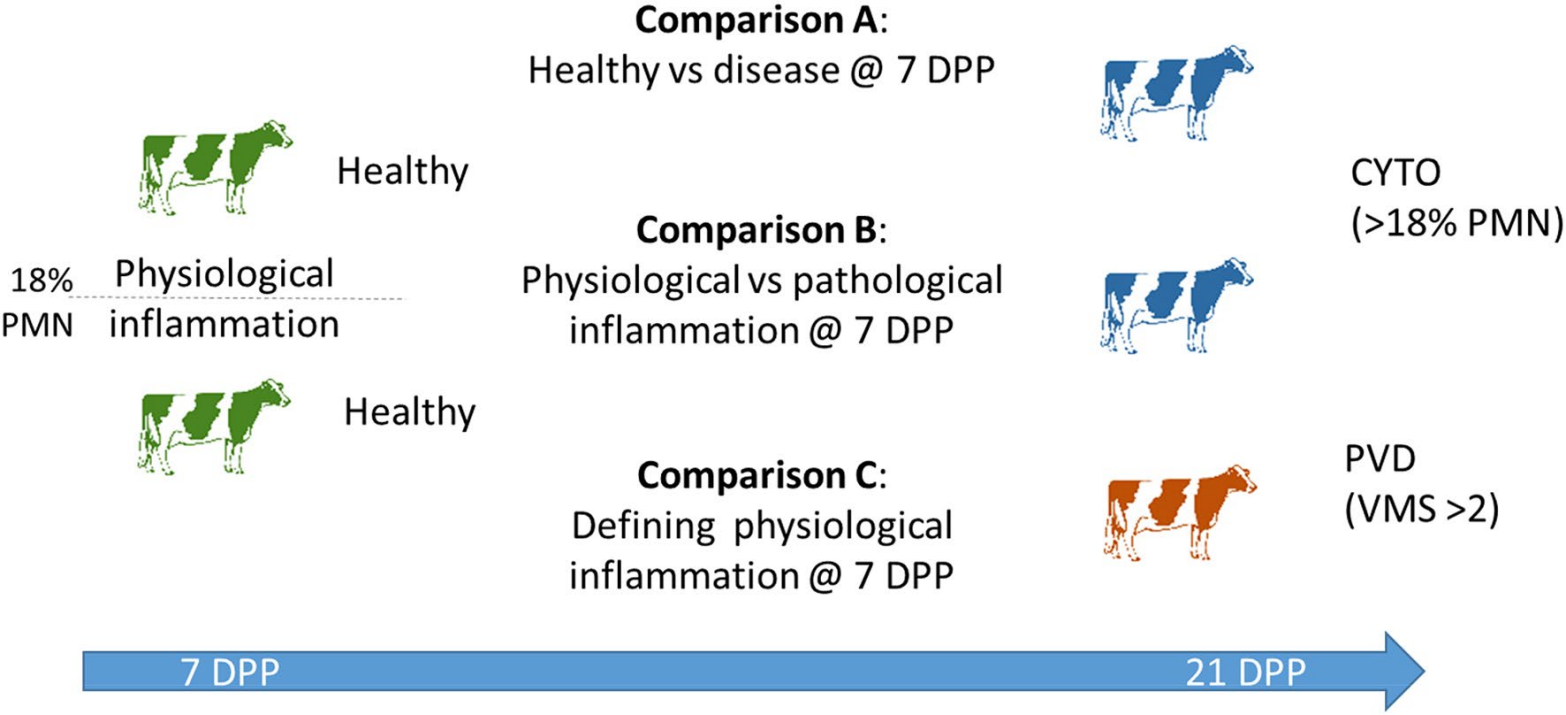
In order to identify potential prognostic biomarkers for the development of uterine disease, RNA-seq was performed on endometrial cells collected at 7 days postpartum (DPP) from 27 cows with subsequent healthy, subclinical (CTYO) or clinical (PVD) disease phenotypes at 21 DPP. Three comparisons were retrospectively performed: (**A**) Cows which remained healthy (n = 10) versus those which developed CYTO (n = 9) and PVD (n = 8) (**B**) Cows with inflammation at 7 DPP which subsequently resolved (n = 5) versus cows which developed CYTO (n = 4; PMN > 18%) (C) Cows which had inflammation at 7 DPP (> 18% PMN) which subsequently resolved (n = 5) versus cows without inflammation (< 18% PMN) which also remained healthy (n = 5).

From the 112 cattle originally sampled, uterine cytobrush samples were chosen from 27 cows on the basis of RNA quality and excluding cows with metritis (vaginal mucus score (VMS) > 2 at 7 DPP) for RNA-sequencing. The final dataset consisted of 10 healthy cows; 9 cows were diagnosed with CYTO on the basis of uterine cytology (PMN ≥ 18%); and 8 cows were diagnosed with PVD (VMS > 2) (Fig. 1). A further independent panel of 51 samples from the original 112 cows were also used for qPCR analysis and consisted of 13 healthy cows, 12 cows with CYTO and 26 cows with PVD (18 of which were also diagnosed with CYTO). All RNA-seq and qPCR was performed retrospectively on endometrial cellular RNA from 7 DPP using the disease classification at 21 DPP. Classification of all animals used for RNA-seq and qPCR can be found in Supplementary Table S1. Summary statistics for all mRNA libraries used for RNA-seq can be found in Supplementary Table S2.

### Comparison A: Differences in gene expression 7 DPP associated with clinical PVD or subclinical CYTO

**Figure 2:**
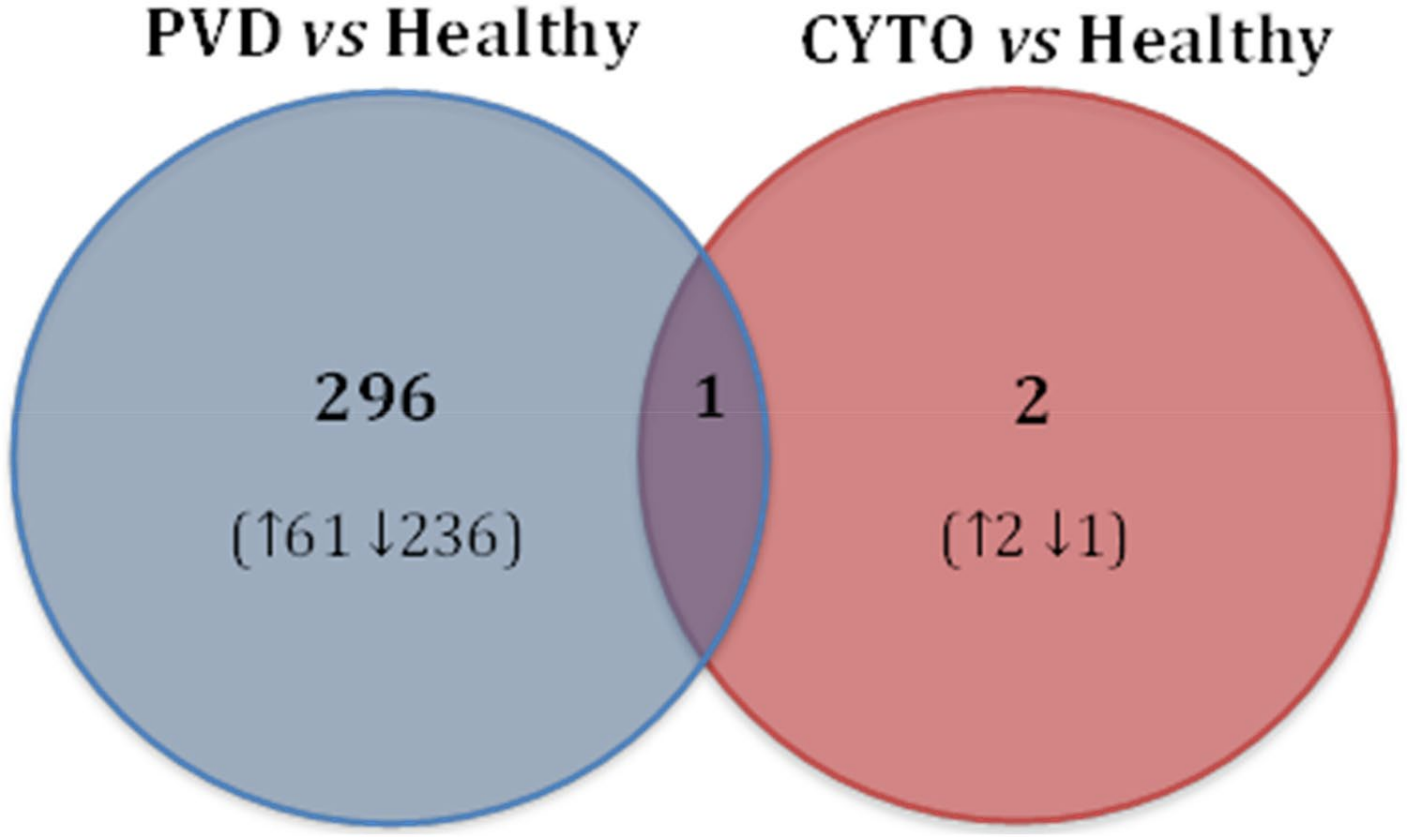
Number of differentially expressed genes in the endometrium at 7 days postpartum (DPP) in cows that developed uterine disease (either PVD or CYTO) relative to cows that remained healthy. Gene expression was determined by RNA-seq using an adjusted *p* < 0.1 and FC > 1. The direction of fold change is represented by arrows in cows which developed uterine disease relative to cows which remained healthy. The number in the centre represents the genes common to both comparisons. Disease diagnosis was performed at 21 DPP by cytology and vaginal mucus score, which diagnosed (CYTO) and purulent vaginal discharge (PVD), respectively. Cows were classified into healthy (n = 10), PVD (n = 8) and CYTO (n = 9) groups.

**Figure 3:**
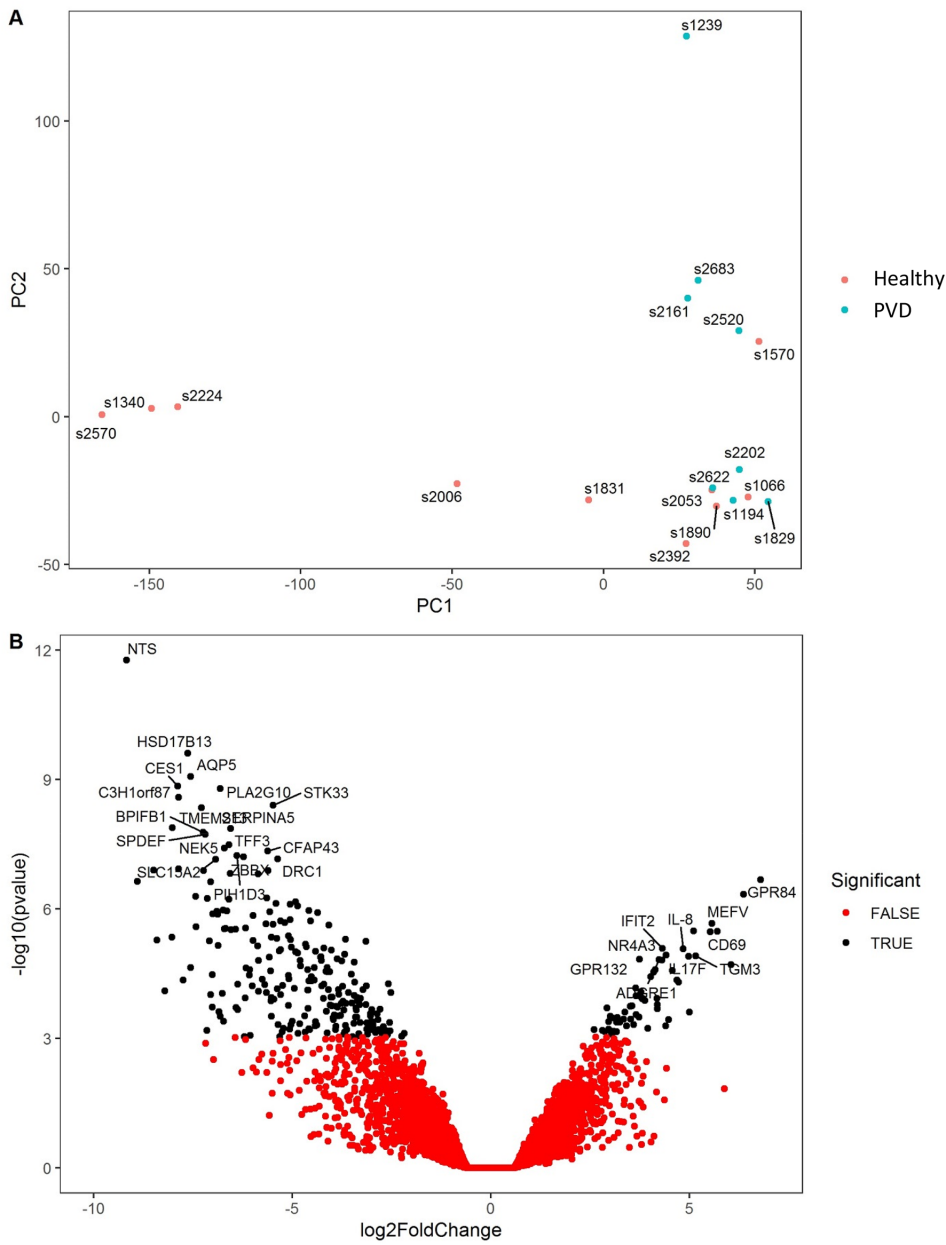
Differentially expressed genes in the endometrium at 7 DPP in cows that developed PVD (n = 8) relative to cows that remained healthy (n = 10). (**A**) PCA plot shows distribution of RNA-seq samples, where colours indicate the two groups and numbers refer to specific cow IDs (as detailed in Supplementary Table S1). (**B**) Gene expression data is presented as a volcano plot using log values of the fold change and *p* value. Each data point represents a single gene, with those in black representing genes that survived the cut off thresholds of adjusted *p* < 0.05 and FC > 1. The 100 most significant differentially expressed genes are labelled, where gene names are available.

Using stringent statistical filtering criteria, 297 genes were identified as significantly differentially expressed at 7 DPP (adjusted *p* value < 0.05) between cows which remained healthy and cows which subsequently developed PVD (Fig. 2). Segregation is evident between samples in both groups as illustrated on the PCA plot (Fig. 3A). Interestingly, the vast majority of these genes (236) were decreased relative to healthy controls and 61 were significantly increased in cows that subsequently developed PVD. The skewed down-regulation of differentially expressed genes (DEG) is evident from the volcano plot (Fig. 3B).

Fold changes of the genes which were increased in healthy cows compared to those which developed PVD varied from 4.5 to 577 fold. A full list of DEG is available in Supplementary Table S3. The ten most significant DEG are also shown in Table 1. The gene with the largest fold change was Neurotensin (*NTS*), which is involved in regulation of the migratory and inflammatory response of macrophages^18^. Multiple members of the same gene family which are also differentially expressed in this dataset are connected to metabolism, cellular enzyme activity and motility. These include the acyl-CoA synthetase medium-chain family member (*ACSM1* and *ACSM3* increased by 16 and 30 fold, respectively); Anterior gradient, protein disulphide isomerase family members *AGR2* and *AGR3* which are involved in steroid metabolism (26 and 29 fold, respectively); Adenylate Kinase genes, *AK7* and *AK9*^19^; Aquaporins (*AQP4*, *AQP5* and *AQP6*) involved in water regulation, were highly increased by over 100 fold in healthy cows. Multiple genes encoding coiled-coil domain-containing proteins (*CCDC13*, *CCDC39, CCDC60, CCDC65, CCDC68, CCDC160, CCDC173* and *CCDC190*) involved in structural integrity of cells are also significantly increased in healthy cows. Similarly, seven gene family members of the Cilia And Flagella Associated Proteins are increased (for full list see Supplementary Table S3). From an immune perspective, one of the most highly differentially increased genes is *CXCL17* (significantly increased by 141 fold). CXCL17 is a major chemotactic factor for macrophages^20^. *CCL28* is significantly upregulated by 17 fold and the CCL28 protein regulates the chemotaxis of cells at several mucosal and epithelial sites, and orchestrates the trafficking and functioning of lymphocytes^21^. The *LF* gene encoding Lactoferrin (also known as lactotransferrin) is significantly increased by 21 fold in cows that do not develop disease. Lactoferrin is a first line mediator in immune defence and response to pathogenic and non-pathogenic injury, as well as a molecule critical for control of oxidative cell function^22^.

**Table 1:** The 10 most significantly differentially expressed genes at 7 DPP in cows that developed PVD. Fold change values represent the change in gene expression in cows which subsequently develop PVD relative to cows which remain healthy. FC = absolute fold change; Adj *p* value—adjusted *p* value. Full gene list is shown in Supplementary file.

Gene symbol	Ensembl ID	Gene name	Function	FC	Adj *p* value
*NTS*	ENSBTAG00000005305	Neurotensin	Involved in smooth muscle contraction and regulation of fat metabolism. Also demonstrates antimicrobial activity	− 577	2.75E−08
*HSD17B13*	ENSBTAG00000031679	Hydroxysteroid 17-Beta Dehydrogenase 13	Involved in lipoprotein metabolism	− 199	1.98E−06
*AQP5*	ENSBTAG00000026813	Aquaporin 5	Water channel protein in the cell membrane. Important role in fluid secretion. Also involved in stabilization and expansion of the E-cadherin adherens junction pathway	− 189	4.56E−06
*CES1*	ENSBTAG00000011021	Carboxylesterase 1	Detoxification of xenobiotics. Possible role in fatty acyl and cholesterol ester metabolism	− 237	5.20E−06
*PLA2G10*	ENSBTAG00000021522	Phospholipase A2 Group X	Calcium-dependent enzyme that hydrolyses phospholipids into fatty acids and other lipophilic molecules. Involved in production of prostaglandins	− 113	5.20E−06
*C3H1orf87*	ENSBTAG00000009481	Chromosome 1 Open Reading Frame 87	Gene Ontology (GO) annotations related to this gene include calcium ion binding	− 233	7.00E−06
*SERPINB4*	ENSBTAG00000039037	Serpin Family B Member 4	Serine protease inhibitor. Possible role in modulation of the host immune response against tumour cells	167	8.61E−06
*TMEM213*	ENSBTAG00000001527	Transmembrane Protein 213	No data currently available	− 156	8.61E−06
*STK33*	ENSBTAG00000011910	Serine/Threonine Kinase 33	Gene Ontology (GO) annotations related to this gene include transferase activity, transferring phosphorus-containing groups and protein tyrosine kinase activity	− 45	8.61E−06
*SERPINA5*	ENSBTAG00000004063	Serpin Family A Member 5	Serine protease inhibitor. Functions as a procoagulant and proinflammatory factor by inhibiting the anticoagulant activated protein C factor	− 94	2.00E−05

Of the 61 significantly upregulated genes in cows which subsequently developed PVD, fold change varied from 6 to 167 fold differential expression. The most significantly enhanced gene was *SERPINB4*, a member of the serpin family of serine protease inhibitors, which are known to play very important roles in uterine function^23^. *SERPINB4* specifically has a recognised role in early inflammation and epithelial barrier dysfunction^24^ and the direction of change is in contrast to another member of the same family, *SERPINA5* which is significantly reduced in expression by a more 94 fold (Table 1). The *NLRP12* gene which encodes the NACHT, LRR and PYD domains-containing protein 12 is upregulated by 18 fold in cows which subsequently develop PVD. These cytoplasmic proteins are involved in pathogen detection and immune response activation. As might be expected in the case of clinical disease, many immune genes are significantly increased which includes the potent IL-1 family members Interleukin 1A and Interleukin 1B (24 and 26 fold, respectively) and the inflammatory protein IL17F (31 fold). The *IL12B* gene which encodes a subunit of interleukin 12 that acts on T and natural killer cells and has a broad array of biological activities is increased in expression by 15 fold. *IL9* is increased by 32 fold and the inflammatory chemokine *CXCL8* is similarly increased by 29 fold.

The top biological process identified by GO for this comparison was cilium movement. The top GO molecular function identified as enriched in this dataset was cytokine receptor binding, reflecting the differential expression of immune genes and which is supported by the KEGG analysis identifying cytokine-cytokine receptor interaction (data not shown).

In contrast to comparing the transcriptome of cows which remained healthy against those which developed PVD, the gene expression differences between healthy cows and cows that subsequently develop CYTO only is less clear. In fact, only 3 genes were identified as significantly differentially expressed (with an adjusted *p* value < 0.05) at 7 DPP (Fig. 2) [full list of DEG in Supplementary Table S3]. Two of these genes were significantly increased in cows which developed CYTO (*LRRN4* and a *Cystatin-9-like* gene) about which little is currently known. The remaining DE gene, Neurotensin (*NTS*), was expressed at a significantly higher level in healthy cows by 105-fold. This gene is involved in the regulation of luteinizing hormone and prolactin release as well as in the inflammatory response of macrophages^18^.

### Comparison B: Physiological inflammation in some healthy cows at 7 DPP obscures detection of inflammatory gene expression profile associated with CYTO

Most cows in this study (78%) had a high level of inflammation (≥ 18% PMN) at 7 DPP (Supplementary Table S1) however a subset of cows (22%) did not exhibit inflammation at this stage. These cows may have avoided early bacterial influx or could potentially have taken longer to develop inflammation and disease. The full list of PMN% for each cow can be found in Supplementary Table S1. The range in PMN% at 7 DPP in cows that remained healthy was 0–66%; whereas in the cows that subsequently developed CYTO it was 0–89%.

**Figure 4:**
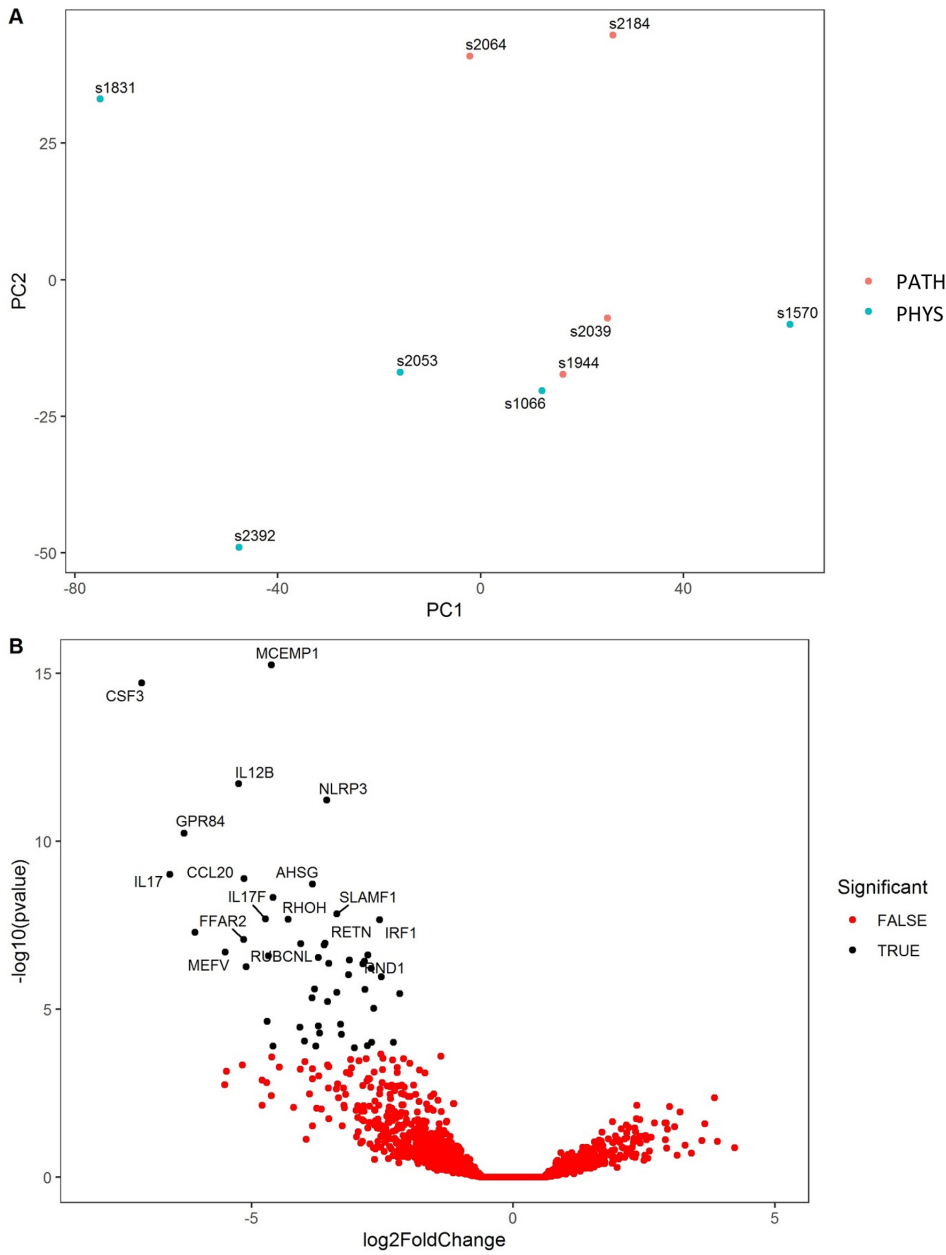
Differentially expressed genes in the endometrium at 7 days postpartum (DPP) in cows with ‘pathological’ inflammation (PATH; ≥ 18% PMN, n = 4) which had developed CYTO by 21 DPP relative to cows with similar levels of inflammation but which remained healthy (PHYS; ≥ 18% PMN, n = 5). A. PCA plot shows distribution of RNA-seq samples, where colours indicate the two groups and numbers refer to specific cow IDs (as detailed in Supplementary Table S1). B. Gene expression data is presented as a volcano plot using log values of the fold change and *p* value. Each data point represents a single gene, with those in black representing genes that survived the cut off thresholds of adjusted *p* < 0.05 and FC > 1. The 20 most significant differentially expressed genes are labelled, where gene names are available.

Therefore, in order to identify DEG that differentiate between cows with physiological inflammation (but remained healthy) and those which subsequently developed CYTO, we excluded cows without inflammation (< 18% PMN) at 7 DPP from this comparison. Comparing cows with physiological inflammation at 7 DPP (i.e. ≥ 18% PMN at 7 DPP) but remained healthy versus those with similar inflammation at 7 DPP but subsequently developed CYTO identified a larger panel of 50 DEG (adjusted *p* value < 0.05). This is reflected in both the PCA plot and volcano plot (Fig. 4A,B). Despite the inflammatory status of all these cows to begin with, expression levels of all 50 genes were significantly enhanced in cows which developed CYTO (full list of DEG in Supplementary Table S3). The top 10 genes by fold change are shown in Table 2. The cows that developed CYTO showed additional clear activation of immune genes, and *CSF3* (Colony Stimulating Factor 3) was increased by 137 fold compared to healthy controls. The immune sensor, NLRP3, was increased by 12 fold and the inflammatory cytokines *IL1A* was increased by 16 fold, as was *IL17* (94 fold) and *IL17F* (27 fold). Other well characterised immune genes were also significantly increased in cows which subsequently develop CYTO including *CD69, CD274, IL12B, CCL20, PTX3, TNFAIP3, CYP27B1, TNF*, *NFKBIA* and *IRF1* (full list of DEG in Supplementary Table S3).

**Table 2:** The 10 most significant differentially expressed genes at 7 DPP in cows with pathological inflammation relative to cows with physiological inflammation. Physiological inflammation was identified as > 18% PMN at 7 DPP but with resolution (< 18% PMN) by 21 DPP whereas pathological inflammation was identified in cows diagnosed with CYTO at 21 DPP (> 18% PMN). Fold change values represent the change in gene expression in cows with pathological inflammation relative to cows which did not develop pathology. FC = absolute fold change; Adj *p* value—adjusted *p* value. Full gene list is shown in Supplementary file.

Gene symbol	Ensembl ID	Gene name	Function	FC	Adj *p* value
*MCEMP1*	ENSBTAG00000004714	Mast Cell Expressed Membrane Protein 1	Thought to be to be involved in regulating mast cell differentiation or immune responses	25	8.38E−12
*CSF3*	ENSBTAG00000021462	Colony Stimulating Factor 3	A cytokine that controls the production, differentiation, and function of granulocytes	137	1.42E−11
*IL12B*	ENSBTAG00000004741	Interleukin 12B	Cytokine that acts on T cells and NK cells. Forms part of IL-23 which promotes production of pro-inflammatory cytokines and stimulates memory T cells. Enhances lytic activity of activated NK cells	38	9.55E−09
*NLRP3*	ENSBTAG00000001273	NLR Family Pyrin Domain Containing 3	Sensor component of the NLRP3 inflammasome, which is required for caspase-dependent maturation and secretion of IL-1β and IL-18. Upstream activator of NFκB	12	2.21E−08
*GPR84*	ENSBTAG00000015592	G Protein-Coupled Receptor 84	Receptor for medium-chain free fatty acid. Potential important roles in various processes from fatty acid metabolism to regulation of the immune system	78	1.70E−07
*IL17*	ENSBTAG00000002150	Interleukin 17	Proinflammatory cytokine produced by activated T cells. Regulates NFκB activity. High levels associated with several chronic inflammatory diseases	94	2.38E−06
*CCL20*	ENSBTAG00000021326	C–C Motif Chemokine Ligand 20	Chemotactic for lymphocytes, and slightly so for neutrophils	35	2.70E−06
*AHSG*	ENSBTAG00000000522	Alpha 2-HS Glycoprotein	Has opsonic properties, promotes endocytosis and influences the mineral phase of bone	14	3.43E−06
*Unknown*	ENSBTAG00000040580	-	-	24	7.72E−06
*SLAMF1*	ENSBTAG00000007927	Signalling Lymphocytic Activation Molecule Family Member 1	Involved in the regulation and interconnection of the innate and adaptive immune response	10	2.17E−05

### Validation of inflammatory gene expression profile in an independent test set

**Figure 5:**
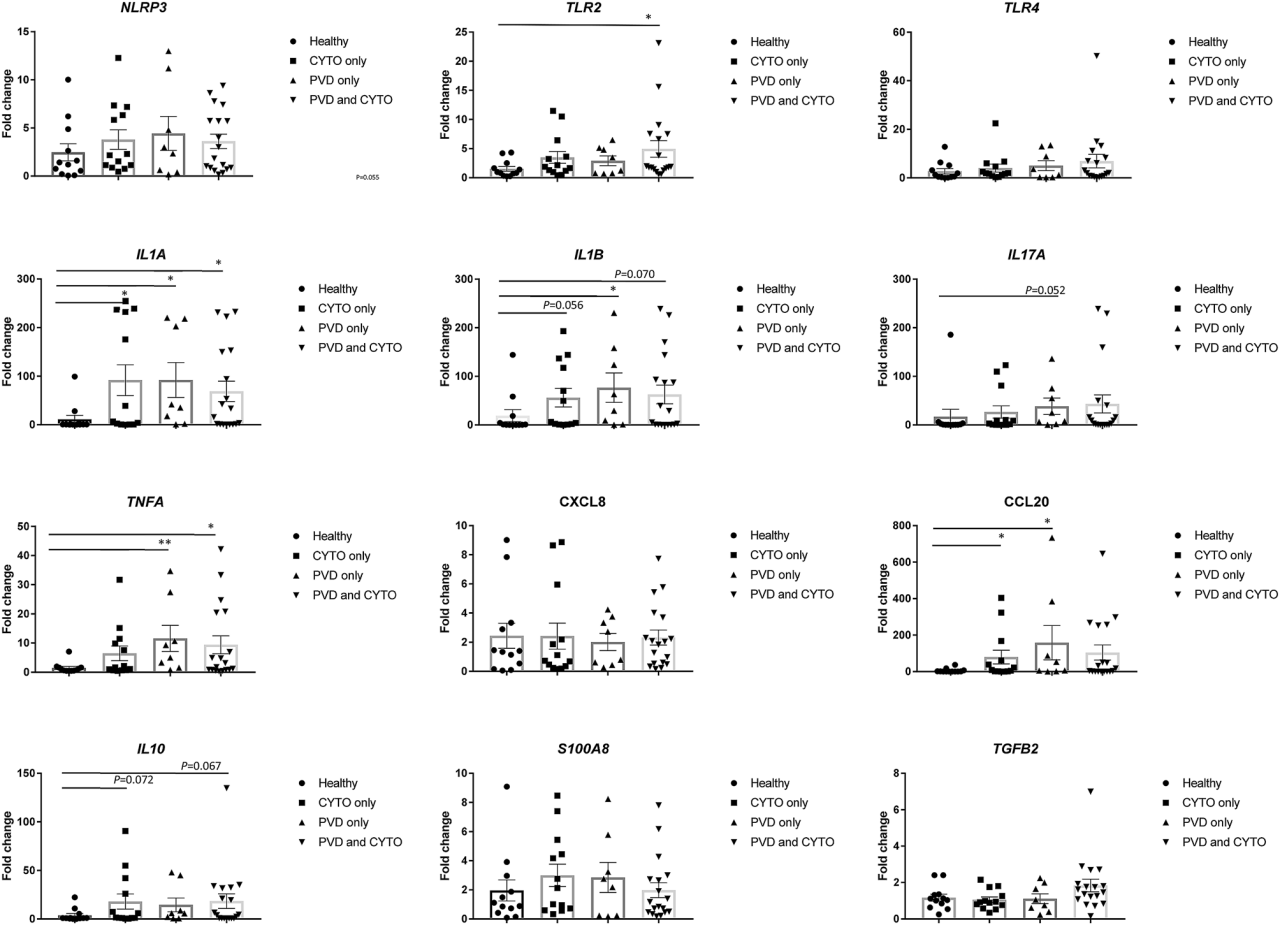
Elevated expression of inflammatory genes 7 days postpartum (DPP) precedes the diagnosis of uterine disease. Quantitative PCR (qPCR) was performed on RNA extracted from endometrial cytobrushes in a validation panel (n = 51) collected at 7 DPP. Disease diagnosis was performed at 21 DPP by cytology and vaginal mucus score, which diagnosed (CYTO) and purulent vaginal discharge (PVD), respectively. Cows were classified into healthy (n = 12), CYTO only (n = 13), PVD only (n = 8) and PVD with CYTO (n = 18) groups. Expression for each of the 12 genes was calculated relative to *RPS15* and *HSP90A1* reference gene expression and fold change is shown relative to the mean of the healthy control group. Bars represent mean ± standard error of the mean (SEM). * p < 0.05.

To evaluate further the reliability of the differential gene expression in an additional and independent panel of endometrial samples, 12 genes were assessed by qPCR in 51 samples at 7 DPP consisting of 12 cows which remained healthy, 13 which subsequently developed CYTO and a further 26 which developed PVD. Eighteen of the cows with PVD also exceeded the diagnostic threshold for CYTO. The 12 genes selected for qPCR analysis were differentially expressed in at least one of the RNA-seq dataset comparisons. Despite a significant degree of inter-animal variation within groups, the general trend in differential expression agreed with that uncovered with the RNA-seq where increases in expression of the inflammatory cytokine *IL17* and anti-inflammatory cytokine *IL10* were detected in the cows which subsequently developed disease (Fig. 5). Expression of Toll-like receptor 2 (*TLR2*) was significantly elevated in cows which developed PVD with the co-occurrence of CYTO. *TNFA* was not significantly elevated in CYTO cows but was significantly increased in cows with PVD. The most highly abundant and significantly differentially expressed were the genes encoding IL-1A; IL-1B and CCL20. *IL1A* was significantly increased by an average of 90.5 fold in CYTO cows, 92 fold in PVD cows and by almost 68 fold in cows which developed both PVD and CYTO. *IL1B* was significantly increased in PVD cows by 75 fold on average. *CCL20* was significantly increased by 79 fold in CYTO cows and almost 157 fold in PVD cows (Fig. 5).

### Comparison C: The resolution of inflammation and restoration of homeostasis in healthy cows

**Figure 6:**
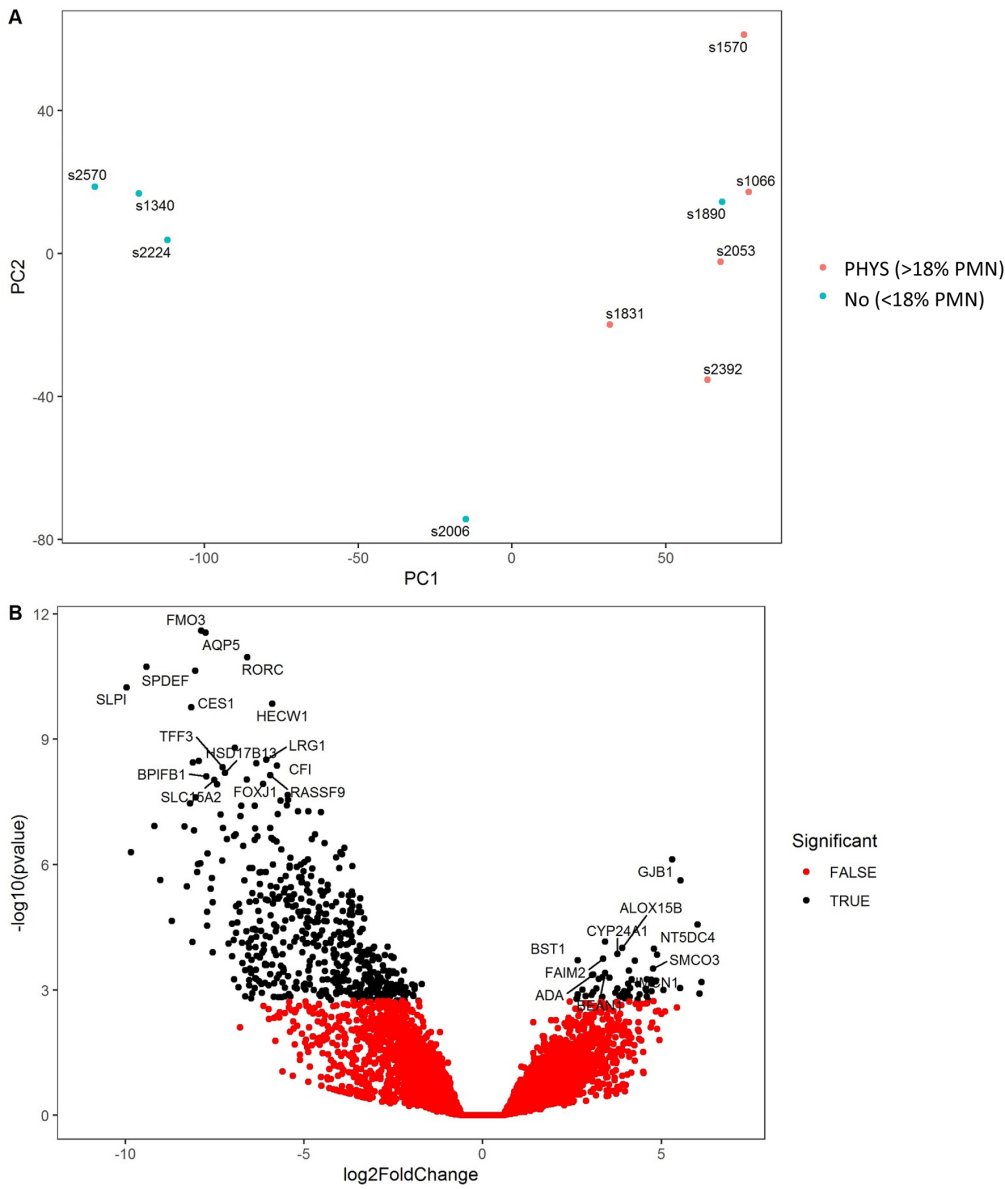
Differentially expressed genes in the endometrium at 7 days postpartum (DPP) in healthy cows with physiological inflammation (n = 5) relative to healthy cows without physiological inflammation (n = 5). (**A**) PCA plot shows distribution of RNA-seq samples, where colours indicate the two groups of cows and numbers refer to specific cow IDs (as detailed in Supplementary Table S1). (**B**) Gene expression data is presented as a volcano plot using log values of the fold change and *p* value. Each data point represents a single gene, with those in black representing genes that survived the cut off thresholds of adjusted *p* < 0.05 and FC > 1. The 30 most significant DEGs are labelled, where gene names are available.

An interesting consequence of splitting the group of healthy cows (diagnosis based on 21 DPP) into those with and without physiological inflammation at 7 DPP described above was that it identified genes potentially involved in physiological inflammation. Comparing endometrial samples from cows above and below the inflammation threshold of 18% PMN, identified 568 differentially expressed genes. The divergent transcriptomic profile between these groups is evident using PCA and volcano plots (Fig. 6).

Interestingly, the vast majority of genes (510) display higher expression levels in the low PMN (no physiological inflammation) group at 7 DPP (see full list of DEG in Supplementary Table S3). Fold changes in expression for genes increased in cows without physiological inflammation varied from 3 to 998 fold, with the highest fold change being secretory leukocyte protease inhibitor *SLPI*, also known as Antileukoproteinase, which protects epithelial tissues from serine proteases^25^. The top 10 DEG are shown in Table 3. Complement component 4 binding protein alpha (*C4BPA*) and the chemokine CXCL17 were increased by 678 fold and 270 fold respectively in cows without signs of physiological inflammation. The vitamin D binding protein gene (*GC)* was increased by 52 fold and Lactotransferrin (*LF*) by 53 fold (Supplementary Table S3).

**Table 3:** The 10 most significantly differentially expressed genes at 7 DPP in cows with and without physiological inflammation. Fold change values represent the change in gene expression in cows with physiological inflammation (defined as > 18% PMN) relative to cows without inflammation (defined as < 18% PMN). FC = absolute fold change; Adj *p* value—adjusted *p* value. Full gene list is shown in Supplementary file.

Gene symbol	Ensembl ID	Gene name	Function	FC	Adj *p* value
*AQP5*	ENSBTAG00000026813	Aquaporin 5	Water channel protein in the cell membrane. Important role in fluid secretion. Also involved in stabilization and expansion of the E-cadherin adherens junction pathway	− 216	2.22E−08
*FMO3*	ENSBTAG00000020597	Flavin Containing Dimethylaniline Monoxygenase 3	Enzyme that catalyses the oxygenation of a wide variety of nitrogen- and sulphur-containing compounds	− 234	2.22E−08
*RORC*	ENSBTAG00000017405	RAR Related Orphan Receptor C	Roles in cellular differentiation, immunity, peripheral circadian rhythm and metabolism	− 96	5.71E−08
*C4BPA*	ENSBTAG00000009876	Complement Component 4 Binding Protein Alpha	Controls activation of the complement cascade through the classical pathway	− 678	7.20E−08
*SPDEF*	ENSBTAG00000021993	SAM Pointed Domain Containing ETS Transcription Factor	Androgen-independent trans-activator of prostate-specific antigen (PSA) promoter	− 263	7.20E−08
*SLPI*	ENSBTAG00000004148	Secretory Leukocyte Peptidase Inhibitor	Secreted inhibitor which protects epithelial tissues from serine proteases	− 998	1.50E−07
*HECW1*	ENSBTAG00000021216	HECT, C2 And WW Domain Containing E3 Ubiquitin Protein Ligase 1	Ubiquitin-protein ligase	− 59	3.15E−07
*CES1*	ENSBTAG00000011021	Carboxylesterase 1	Detoxification of xenobiotics. Possible role in fatty acyl and cholesterol ester metabolism	− 285	3.42E−07
*SLC25A48*	ENSBTAG00000018287	Solute Carrier Family 25 Member 48	No data currently available	− 122	2.83E−06
*LOC407163*	ENSBTAG00000046375	Trappin 5	Host-defense peptide with antiproteolytic, antiinflammatory, and antimicrobial functions	− 277	4.60E−06

For the 58 genes increased in the cows with physiological inflammation, fold change varied between 6 and 70 fold. The highest induced genes encode Fc gamma 2 receptor-like protein. CYP24A1 is an enzyme expressed in the mitochondrion of humans and other species. It catalyses hydroxylation reactions which lead to the degradation of 1,25-dihydroxyvitamin D3 by 14 fold (Supplementary Table S3).

To further investigate the dependency between PMN influx and inflammatory gene expression, a correlation analysis was performed between PMN% and relative mRNA abundance results for the genes assessed by qPCR. In support of the predominant role of the recruited PMN cells in immune gene expression as described above, results showed significant correlations between PMN% and each of these immune genes. A correlation coefficient (r^2^) of 0.69 was detected for IL1A and PMN% whereas it was 0.73 for *CXCL8* (Supplementary Fig. S1). Finally, in order to determine the potential contribution of damage to epithelial cell layer in the uterus to the immune gene expression profile differences detected, the expression of cell-type specific marker genes were extracted from the RNA-seq data. Clear increased expression of epithelial-specific genes (*MUC1*, *KRT18* and *CDH1*) was evident in cows without physiological inflammation whereas in contrast, increased expression of the stromal-specific gene vimentin (*VIM*) was evident in cows with physiological inflammation (Supplementary Fig. S2).

## Discussion

Parturition is a metabolically stressful period for the dairy cow, and the physiological transition from pregnancy to peak milk production while supporting uterine involution requires significant energy reserves^26^. By the time the signs of clinical uterine disease are apparent, considerable development of infection and pathological inflammation (as evidenced by PVD) has occurred which results in the widespread negative consequences on reproduction and milk yield^3,27^. The tissue pathology associated with advanced disease development usually inhibits involution, contributes to a collapse in protective microbial diversity, and disseminates inflammatory signals. In such a scenario of advanced disease, the response of any therapeutic intervention is likely to be limited. Therefore, key to restoration of uterine homeostasis is early and accurate prognosis of the trajectory that leads to disease.

Building on earlier work by our group^12,28^, we tested the hypothesis that comprehensive analysis of the early postpartum uterine immune response could identify potentially maladaptive molecular signals that contribute to dysregulated immunity and subsequent uterine disease. In endometrial cytobrush samples from a total of 78 cows, a combination of RNA-sequencing, complemented with qPCR was used to reconstruct the cellular signalling mechanisms occurring at 7 DPP in endometrial cells from cows which remain healthy or subsequently develop subclinical (CYTO) or clinical (PVD) uterine disease.

Results showed clear activation of inflammatory genes and regulatory networks associated with the subsequent development of PVD. In agreement with previous literature by us and others, significant upregulation of genes encoding the potent Interleukin-1 family of inflammatory cytokines including *IL1A* and *IL1B* were detected^29,30^ signifying an exacerbation of inflammation in cows which subsequently develop clinical uterine disease. One of the added advantages of the RNA-seq approach undertaken here is the quantitative read outs that illustrates a predominant role for *IL1B* in the activation of this response in cattle. While related studies have examined the role of IL-1A in uterine disease in cattle in detail^31^, here we show that another IL-1 family member, *IL1B* was one of the top differentially expressed genes showing the priority to which the activation of an immune response was given in these cells. As both IL-1α and IL-1β have been shown to recruit different innate cells—accumulation of IL-1α correlated with the infiltration of neutrophils, and the expression of IL-1β correlated with later migration of macrophages, this could have important consequences for uterine disease pathogenesis^32^.

Another advantage of the RNA-seq approach is the added detail which accrues from the pan-genomic analysis of the entire transcriptome. The activation of cellular receptors, for example, sheds light not only on disease pathogenesis but also identifies potential therapeutic targets. Inflammasome complexes are attracting a lot of recent attention for the role they play in inflammatory disease and for the potential they offer as novel therapeutic targets. Results here show increased expression of the Nod like receptor gene *NLRP12*, which regulates NF-kB signalling and inflammasome activation^33^. Chemokine (C–C motif) ligand 20 (*CCL20*) or Macrophage Inflammatory Protein-3 (*MIP3A*) which is strongly chemotactic for lymphocytes and weakly attracts neutrophils^34^ was significantly upregulated and also validated by qPCR. Not much is known about the role of CCL20 in cattle, but human gene expression of CCL20 can be induced by microbial factors such as lipopolysaccharide (LPS), and inflammatory cytokines such as tumour necrosis factor^35^. This chemokine has a number of functions potentially relevant to bovine reproduction including in the coordination of signals between the ovary and the uterus as well as the chemotactic response to sperm^36^. Circulating levels in serum are attracting recent interest in human studies^37^, and its significantly elevation in response to post partum disease warrants further investigation.

Interestingly however, the vast majority of DEG in this comparison were decreased in cows which subsequently developed PVD relative to cows which remained healthy. Results show a clear upregulation of genes involved in the maintenance of cellular functions in healthy cows, which collectively play a role in the progress of involution. The evidence above suggests that this process is arrested in cows which subsequently develop disease and a reprioritisation toward the activation of an immune response is evident. This is further reflected by ‘cilium movement’ being the top biological process identified by GO. Bovine endometrial cells have microvilli^38^, and their restoration may explain the differential expression of many of these genes^39^ during uterine involution and decidualisation. One of the genes increased in cows which remained healthy, and which has the highest expression level was the gene encoding lactoferrin (*LF* also known as lactotransferrin, LTF). In addition to its potent antimicrobial properties, lactoferrin can bind and sequester lipopolysaccharides, thus is a negative regulator of the inflammatory response and may be an important target to help restore uterine homeostasis^40^.

The inflammatory profile associated with the subsequent development of sub-clinical uterine disease (CYTO) was obscured somewhat by the presence of physiological inflammation in the majority of cows at 7 DPP. Therefore, we suspected that the low numbers of genes differentially expressed at 7 DPP between all cows that remained healthy and those that subsequently developed CYTO may reflect the presence of variable levels of physiological inflammation between these cows. Significantly divergent cellular profiles in terms of the ratio of PMN to epithelial cells between cows may obscure the detection of a putative marker of subsequent disease development. Despite this, direct comparisons between cows with physiological inflammation which subsequently developed disease did identify an inflammatory response, with increased expression of *TNFA* as well as *CCL20*. Expression of the inflammatory cytokines *IL17A* and *IL17F* were also enhanced in cows which develop CYTO compared to those which remained healthy. A consistent role for *IL1B* reflected the DEG profile detected with PVD; and this was validated by qPCR. In agreement with the RNA-seq data and despite a significant degree of inter-animal variation within groups, it is clear that a core inflammatory gene expression profile at 7 DPP is associated with the subsequent development of both clinical and sub-clinical uterine disease.

Cows with and without evidence of physiological inflammation at 7 DPP (over 500 significantly DEG) had a very distinctive transcriptome at 7 DPP and this dataset yielded interesting insights into the potential mechanisms for the regulation of inflammation in the post-partum uterus. Again reflecting the comparison between healthy and PVD cows, transcription of the majority of the genes was increased in non-inflamed cows, suggesting that active tissue repair processes are underway^28^. One of the highest DEG in cows which remained healthy was the gene encoding secretory leukocyte protease inhibitor (SLPI), which is an enzyme that protects epithelial tissues from serine proteases^25^. Similarly the chemokine *CXCL17* and vitamin D binding protein gene (*GC)* are also both significantly increased. Again, the Lactotransferrin gene (*LF*) is both highly expressed and significantly increased indicating a predominant role in ameliorating inflammation in these cells. The top significantly enriched molecular function as defined by GO was *cytokine activity* and top enriched biological process was identified as the *regulation of the inflammatory response*. The top enriched pathway identified by KEGG was the *IL-17 signalling pathway*. Relevantly, we detected high correlations in these cows between PMN% and inflammatory gene expression indicating that it is the influx of immune leukocytes that are driving the inflammatory response detected.

Interestingly, cows without inflammation at 7 DPP also appeared to have a more intact endometrial epithelial barrier. Increased expression of epithelial-specific genes (*MUC1*, *KRT18* and *CDH1*) was evident in cows without physiological inflammation whereas in contrast, increased expression of the stromal-specific gene vimentin (*VIM*) was evident in cows with physiological inflammation. The epithelial cell layer is often damaged in the early postpartum period due to difficulties at calving^41^ and consequently many of the risk factors that precede the development of uterine disease are associated with endometrial damage, such as dystocia, twinning, primiparity and the gender and size of the calf. The importance of an intact endometrial epithelial barrier has been emphasised by recent histological analysis of uterine samples from cows with metritis, which had lost a large amount of the luminal epithelium in inter-caruncular areas of the endometrium in comparison to healthy cows^42^. Damage of the endometrial epithelium also allows the entry of opportunistic bacteria such as *T.pyogenes*, which cannot usually penetrate the intact epithelium due to a lack of cholesterol^43^. It may be that cows without inflammation had a more robust barrier, possibly due to an easier calving, or that greater endometrial damage occurred in cows with physiological inflammation due to higher levels of inflammatory-associated tissue damage. This comparison gives a unique insight often overlooked in other studies, where all ‘healthy’ cows are grouped together regardless of their early inflammatory status. This data suggests that the active regulation of physiological inflammation and the restoration of uterine homeostasis is a function of the resident epithelial cells.

Sampling cows with natural uterine infection presents a number of logistical challenges, namely the inter-animal variation in stage of disease which results in significant data heterogeneity. Some of that heterogeneity is due to the complex presentation of PVD, which can occur in combination with CYTO (as occurred in 63% of the PVD cows in this study). Although a clear additive effect of both conditions on the proportion of cows not pregnant up to 200 days after parturition in over 1000 Holstein cows has been reported^5^, the precise effects of divergent conditions on the inflammatory response requires additional in-depth future analysis. Despite the heterogeneity, we have uncovered a clear pro-inflammatory immune response at the transcriptomic level in cows which develop both clinical and sub-clinical uterine disease. Here we showed how accounting for physiological inflammation yields superior resolution of pathogenic immune changes. A potential limitation of this study is the absence of the valuable insights that could be gained from parallel analysis of the microbiome in the same cows. Recent analysis of the uterine microbiome in Irish dairy cows from 3 farms has shown that reduced microbial diversity at 7 days postpartum is associated with the development of PVD at 21 DPP^44^, and it would be of interest to examine the contribution of a dysbiotic microbiome to the uterine inflammatory profile. An additional potential caveat of the work described is that a number of cows will resolve disease diagnosed at 21 DPP, and this may not negatively impact on subsequent fertility, and therefore later diagnosis could result in a specific biomarker. However the diagnosis of CYTO was performed within the peak PMN window shown in other studies (between 10 and 24 DPP)^45^ and our recent work did detect negative fertility consequences in pasture-basted dairy cows diagnosed with PVD at 21 DPP^6^. Furthermore, uterine inflammation has recently been identified as a key parameter contributing to divergent reproductive outcomes in dairy cows with high or low genetic merit for fertility^46^. In seasonal based dairy systems, where a tight calving to conception period of 85 days is required, even moderate sub-fertility due to delayed resolution of inflammation could potentially have significant consequences for system sustainability.

## Conclusion

While inflammation is a core component of the immune response and indeed has a normal physiological function required for the restoration of homeostasis postpartum^14^, there is now a clear consensus emerging from the literature that dysregulated inflammation contributes to uterine disease^16,47^. Our analysis has identified components of a core inflammatory gene set indicative of a shift from physiological to pathological inflammation. This shift is governed by both qualitative and quantitative changes in inflammatory genes, specifically in the IL-1 pathway. We have also identified a number of potential therapeutic target molecules including lactotransferrin and the chemokine CCL20. The putative gene expression biomarkers can now be validated at the protein level in in samples from cows on additional farms. These findings complement our on-going efforts to identify biomarkers of uterine disease in blood^48^ or in vaginal mucus^49,50^ which will ultimately improve both disease diagnosis and therapeutic intervention.

## Methods

### Animals and sample collection

Sample collection was performed on a commercial, spring-calving dairy farm in Ireland with an average milk yield of 6417 L/year. Cattle were mixed-parity Holstein-Friesians and were calved indoors. Approximately 70% of the herd were artificially inseminated. Cows which experienced difficult deliveries (calving assistance, twins), vulva sores and pneumonia were not included in the study. Samples were collected from 112 cattle in the morning after milking at 7 and 21 days postpartum. Cytobrush collection was performed as previously described^51^. Briefly, this involved trans-cervical insertion of a double guarded cytobrush into the uterus. Once the cervix was reached, the inner brush was pushed through the outer guard and rotated clockwise along the wall of the uterus three times to harvest the cells. The cytobrush was then reinserted into the inner guard and removed and stored in a labelled cryotube on dry ice. All tubes stored on dry ice were transferred to the − 80 °C freezer on return to the laboratory. A second inner cytobrush was inserted whilst the outer guard was still in place which enabled the collection of cells from the same position within the uterus. The second cytobrush was rolled anticlockwise on a glass microscope slide, and later stained with Diff-Quik (a modified Wright Giemsa stain) and used to classify the cows cytologically.

### Ethical approval

All procedures described were conducted under ethical approval and experimental license from the Irish Health Products Regulatory Authority (licence number AE19132/038) in accordance with the Cruelty to Animals Act 1876 and the European Communities (Amendments of the Cruelty to Animals Act 1876) Regulations, 1994.

### Cytology and PVD diagnosis

Once the cytology slides had been stained and dried, they were classified by observing the number of PMNs and epithelial cells present. The slides were graded blind without knowledge of sample ID or background. A total of 200 cells were counted at 400×magnification. The animals were classified in line with recent publications, using the cut off of 18% PMN at 21 DPP^51,52^. Those with ≤ 18% PMN at 21 DPP were classified as healthy, whilst cows with > 18% PMN at 21 DPP were diagnosed with cytological endometritis.

A vaginal mucus score (VMS) was recorded for each cow at 7 and 21 DPP. Assessment of vaginal mucus was carried out using a gloved hand. A scoring system of 0–3 was used, as previously described^53^. Briefly, a score of 0 or 1 was given to vaginal discharge that was clear or contained flecks of pus and the cow was classified as healthy. A score of 2 or 3 was given to samples with over 50% purulent or muco-purulent material present in the vaginal exudate and the cow was diagnosed with purulent vaginal discharge. A proportion of cows presented with PVD also had a concurrent CYTO diagnosis, as shown in Supplementary Table S1. Cattle were classified by endometrial cytology score at 7 and 21 DPP and the vaginal mucus score at 21 DPP (Supplementary Table S1). Cows that potentially had clinical metritis at 7 DPP (VMS > 2) were excluded from subsequent analysis to increase the reliability of the prognostic gene expression profile for disease.

### RNA extraction

Endometrial cytobrushes were placed in 350 µL of TRIzol reagent and vortexed vigorously for 1 min to lyse the cells. Following incubation at room temperature for 5 min, chloroform was added to each sample, shaken vigorously for 15 s and centrifuged at 12,000×*g* for 15 min at 4 °C. The aqueous phase was transferred to a new tube. Ethanol (70%) was added to the aqueous phase, mixed immediately, and then transferred to an RNeasy spin column. The RNA extraction was completed using the RNeasy kit (Qiagen Ltd., Crawley, UK), following the manufacturer’s instructions. This included a number of wash steps using the kit buffers supplied, and finally elution in 30 µL of RNase-free water. The quantity and quality of RNA present in the samples was determined using the NanoDrop ND-1000 UV–Vis Spectophotometer (NanoDrop Technologies Inc., Wilmington, DE, USA) and 2100 Agilent Bioanalyzer respectively.

### Library preparation and sequencing

A total of 30 samples were chosen for RNA sequencing, based on cow classification and RNA quality (RIN > 6.5). Three of these samples were later excluded as the cows had VMS scores ≥ 2, suggesting that they may have had clinical metritis. All samples used were acquired at 7 DPP. Library preparation was carried out using the Illumina TruSeq Stranded mRNA Library Prep Kit, to convert mRNA into cDNA libraries for DNA sequencing. Briefly this involved isolation of poly-A tailed mRNA using poly-T oligo attached magnetic beads, reverse transcription to form double stranded cDNA and the ligation of indexing adaptors. Indexes were allocated to specific samples prior to library construction so that each sample within a pool had a unique bar code. Following adapter ligation, DNA fragments were selectively enriched by performing PCR. The number of PCR cycles was reduced from the 15 cycles recommended in the manufacturer’s protocol to 12 following optimisation on practice samples. Quality control checks were performed to assess the quality and quantity of the ds cDNA libraries. The Agilent 2100 Bioanalyzer (Agilent Technologies) was used to assess purity of the samples, using the Agilent DNA 1000 kit. Library quantity was measured using the Qubit fluorometer. Five pools of 6 multiplexed samples were constructed, and the indexed libraries were sent to Clinical Genomics (Toronto, Canada) where they were sequenced using an Illumina HiSeq 2500, to produce 100 bp single-end reads.

### RNA-seq data analysis

Quality assessment of the raw sequence data was carried out using the software FastQC (v 0.1.5; https://www.bioinformatics.babraham.ac.uk/projects/fastqc/). Data was quality and adapter trimmed using the BBDuk java package to trim Illumina adapter sequences and any low quality bases (Phred score < 20) from the 3′ end of sequence read pairs. Reads were aligned to the bovine genome UMD3.1 using the Spliced Transcripts Alignment to a Reference (STAR) aligner^54^. A maximum of two mismatches with the reference genome were allowed and only uniquely mapped read pairs were retained for downstream analysis. Read counts overlapping all protein coding genes in the UMD3.1 Ensembl (v.86) annotation were estimated using featureCounts^55^. To filter out lowly expressed genes, genes with less than one count per million in at least five samples were discarded from the analysis. Remaining gene counts were normalized uses the median of ratios method as implemented in DeSeq2 (version 1.18.1)^56^ to account for varying sequencing depth between samples. Transcript counts were modelled by fitting the data to a negative binomial distribution using genewise dispersion estimates and differentially expressed genes were identified with a generalized linear model likelihood ratio test. Statistical tests were corrected for multiple testing using the Benjamini–Hochberg method. DE genes with an adj. *p* value < 0.05 and a log_2_ fold change threshold of 1 were used for further DE gene data exploration and pathway analysis.

### Quantitative real-time PCR validation

Endometrial cytobrush RNA acquired at 7 DPP from additional animals within the original group of 112 cows was used for qPCR validation. Samples of inadequate RNA quality (RIN value < 6.5) or quantity were not included, resulting in the analysis of 51 samples by qPCR. Total RNA (1 µg) from each cytobrush was reverse transcribed using the High Capacity cDNA Reverse Transcription Kit (Life Technologies Inc., Carlsbad, CA, USA) according to the manufacturer’s instructions. Following reverse transcription, cDNA were diluted to 5 ng/µl with nuclease-free H_2_O. Quantitative PCR analysis was performed in triplicate using 10 ng of cDNA in 96-well plates using the Fast SYBR Green Master Mix (Life Technologies Inc.). A no-template control and minus reverse transcriptase (RT) were used to check for non-specific amplification. Twelve immune genes were selected for validation of the RNA-seq results, which were chosen due to their involvement in many of the top differentially regulated pathways and functions. Intron-spanning, gene-specific primers were designed using the primer-blast tool on the NCBI website (www.ncbi.nlm.nih.gov/tools/primer-blast) and are listed in Supplementary Table S4. The amplification efficiency for each gene was tested using a serial dilution of pooled cDNA from the tissue of interest. Efficiencies were between 80 and 104% for all genes (data not shown). Where efficiencies were less than 90%, the fold change calculation was modified to reflect the calculated efficiency. Adjustments were performed to account for inter-plate variation using the inter-plate calibrator sample included on all plates. Four reference genes were tested (*GUSB, SDHA, HSP90AB1* and *RPS15*), which were selected from the RNA-seq dataset for their stability (i.e. they were not differentially expressed). Genorm analysis software was used to determine the average expression stability (M) value for each gene, and the two genes that were most stable (*HSP90AB1* and *RPS15*) were used for normalisation. Fold changes were calculated using the delta delta Ct method^57^. Statistical analysis of qPCR Ct data was performed on delta Ct values using a Kruskal–Wallis ANOVA in Graphpad Prism 5 software after testing for normality. A *p* value of < 0.05 was taken as statistically significant.

## Supplementary information

Supplementary Information 1.

Supplementary Information 2.

Supplementary Information 3.

## Data Availability

The datasets generated and analysed during the current study are available in the GEO repository, [accession number GSE125964]. GEO data repository, at https://www.ncbi.nlm.nih.gov/geo/query/acc.cgi?acc=GSE125964.
