# Supplementary material for: Qualitative and quantitative differences in endometrial inflammatory gene expression precede the development of bovine uterine disease
Source: Sci Rep. 2020 Oct 26;10:18275. doi: 10.1038/s41598-020-75104-7 (PMC7588428; doi:10.1038/s41598-020-75104-7)
Supplement: Supplementary file 3 — Supplementary Information 3. [file 41598_2020_75104_MOESM3_ESM.docx]

Qualitative and quantitative differences in endometrial inflammatory gene expression precede the development of uterine disease

Amy Brewer^1, 3^, Paul Cormican^1^, Joseph J Lim^2^, Aspinas Chapwanya^2^_,_ Cliona O’ Farrelly^3,4^ and Kieran G. Meade^1^*

**Supplementary Information**

**Supplementary Table S1.** Animal classification by inflammatory and disease status. Cows (n=112) were classified on the basis of both percentage polymorphonuclear leukocyte (PMN) infiltration assessed by cytology and vaginal mucus score (VMS) at 7 and 21 days postpartum (DPP). Results are shown in the table below for 78 cows that were deemed eligible for analysis (after cows with metritis and samples with technical issues like low RNA quality were removed). Scoring at 21 DPP determined the health status of each cow at this time point. Grey shading indicates cattle that were selected for RNA-seq.

| Cow ID | Group | Lactation | PMN % | PMN % | VMS D7 | VMS D21 | Health status D21 |  |
| --- | --- | --- | --- | --- | --- | --- | --- | --- |
|  |  |  | D7 | D21 |  |  |  | Application |
| 2392 | Healthy | 2 | 60 | 1 | 0 | 0 | Healthy | RNAseq |
| 1570 |  | 7 | 43.5 | 1 | 1 | 0 | Healthy | RNAseq |
| 1831 |  | 5 | 36 | 0 | 1 | 1 | Healthy | RNAseq |
| 1066 |  | 10 | 53.5 | 2.5 | 0 | 0 | Healthy | RNAseq |
| 2053 |  | 4 | 32.5 | 6.5 | 0 | 1 | Healthy | RNAseq |
| 1641 |  | 6 | 29 | 7 | 0 | 1 | Healthy | qPCR |
| 1761 |  | 6 | 21 | 1.5 | 0 | 0 | Healthy | qPCR |
| 2361 |  | 2 | 35.5 | 17.5 | 1 | 0 | Healthy | qPCR |
| 2676 |  | 1 | 39 | 2.5 | 0 | 0 | Healthy | qPCR |
| 1733 |  | 6 | 41.5 | 12 | 0 | 0 | Healthy | qPCR |
| 2055 |  | 4 | 66 | 12 | 0 | 1 | Healthy | qPCR |
| 1472 |  | 7 | 38.5 | 12.5 | 0 | 0 | Healthy | qPCR |
| 1340 | Healthy | 8 | 2.5 | 1 | 0 | 1 | Healthy | RNAseq |
| 2006 |  | 4 | 6.5 | 0.5 | 0 | 0 | Healthy | RNAseq |
| 2570 |  | 1 | 0 | 1 | 0 | 0 | Healthy | RNAseq |
| 1890 |  | 5 | 6.5 | 10.5 | 0 | 0 | Healthy | RNAseq |
| 2224 |  | 3 | 5 | 0.5 | 0 | 0 | Healthy | RNAseq |
| 1487 |  | 7 | 4 | 7.5 | 1 | 0 | Healthy | qPCR |
| 2241 |  | 3 | 0.5 | 0.5 | 1 | 1 | Healthy | qPCR |
| 2409 |  | 2 | 10.5 | 17.5 | 1 | 0 | Healthy | qPCR |
| 2597 |  | 2 | 7.5 | 10 | 0 | 1 | Healthy | qPCR |
| 2636 |  | 1 | 1 | 4 | 0 | 1 | Healthy | qPCR |
| 2064 | CYTO | 4 | 57 | 73 | 0 | 1 | CYTO | RNAseq |
| 2184 |  | 3 | 76 | 57.5 | 0 | 0 | CYTO | RNAseq |
| 2039 |  | 4 | 87 | 85 | 0 | 1 | CYTO | RNAseq |
| 1944 |  | 5 | 53.5 | 62 | 0 | 1 | CYTO | RNAseq |
| 1498 |  | 7 | 24 | 76 | 0 | 0 | CYTO | qPCR |
| 1984 |  | 4 | 65 | 33.5 | 0 | 0 | CYTO | qPCR |
| 1985 |  | 4 | 25 | 24.5 | 1 | 0 | CYTO | qPCR |
| 2254 |  | 3 | 32.5 | 68.5 | 0 | 1 | CYTO | qPCR |
| 2727 |  | 1 | 40 | 45 | 0 | 0 | CYTO | qPCR |
| 912 |  | 11 | 57.5 | 35.5 | 0 | 1 | CYTO | qPCR |
| 1698 |  | 6 | 30 | 75 | 0 | 1 | CYTO | qPCR |
| 2684 |  | 1 | 42.5 | 60 | 1 | 0 | CYTO | qPCR |
| 2566 |  | 1 | 75.5 | 67.5 | 0 | 1 | CYTO | qPCR |
| 2673 |  | 1 | 52 | 23 | 1 | 1 | CYTO | qPCR |
| 2686 |  | 1 | 89 | 78.5 | 0 | 1 | CYTO | qPCR |
| 2421 | CYTO | 2 | 14.5 | 59.5 | 0 | 1 | CYTO | RNAseq |
| 1183 |  | 9 | 2 | 53 | 0 | 0 | CYTO | RNAseq |
| 2167 |  | 3 | 0 | 45 | 0 | 1 | CYTO | RNAseq |
| 2382 |  | 2 | 16 | 55 | 0 | 1 | CYTO | RNAseq |
| 2178 |  | 4 | 3.5 | 28.5 | 0 | 0 | CYTO | RNAseq |
| 1738 |  | 6 | 7 | 20 | 0 | 1 | CYTO | qPCR |
| 2436 |  | 2 | 16 | 18.5 | 0 | 1 | CYTO | qPCR |
| 2202 | PVD | 3 | 53 | 58 | 0 | 3 | CYTO + PVD | RNAseq |
| 1239 |  | 9 | 74.5 | 33 | 1 | 3 | CYTO + PVD | RNAseq |
| 2161 |  | 3 | 52.5 | 71.5 | 1 | 2 | CYTO + PVD | RNAseq |
| 1829 |  | 5 | 86.5 | 89 | 0 | 2 | CYTO + PVD | RNAseq |
| 1467 |  | 7 | 60.5 | 24 | 0 | 3 | CYTO + PVD | qPCR |
| 1685 |  | 6 | 56.5 | 65.5 | 0 | 3 | CYTO + PVD | qPCR |
| 1855 |  | 5 | 21.5 | 62 | 0 | 3 | CYTO + PVD | qPCR |
| 2211 |  | 3 | 72 | 21 | 0 | 3 | CYTO + PVD | qPCR |
| 2240 |  | 3 | 31 | 86 | 0 | 2 | CYTO + PVD | qPCR |
| 2245 |  | 3 | 36.5 | 36.5 | 0 | 2 | CYTO + PVD | qPCR |
| 2598 |  | 1 | 41.5 | 29 | 0 | 2 | CYTO + PVD | qPCR |
| 2602 |  | 1 | 33.5 | 40.5 | 0 | 3 | CYTO + PVD | qPCR |
| 2629 |  | 1 | 45 | 69.5 | 0 | 3 | CYTO + PVD | qPCR |
| 2639 |  | 1 | 62.5 | 76 | 0 | 3 | CYTO + PVD | qPCR |
| 1871 |  | 5 | 57.5 | 57 | 1 | 3 | CYTO + PVD | qPCR |
| 2213 |  | 3 | 64.5 | 67.5 | 0 | 3 | CYTO + PVD | qPCR |
| 2381 |  | 3 | 57 | 86 | 0 | 2 | CYTO + PVD | qPCR |
| 2443 |  | 2 | 71 | 40 | 1 | 3 | CYTO + PVD | qPCR |
| 1887 |  | 5 | 70 | 93 | 0 | 3 | CYTO + PVD | qPCR |
| 1510 |  | 7 | 77.5 | 90.5 | 0 | 3 | CYTO + PVD | qPCR |
| 2590 |  | 1 | 87.5 | 92.5 | 0 | 3 | CYTO + PVD | qPCR |
| 1194 | PVD | 9 | 31 | 6.5 | 0 | 2 | PVD only | RNAseq |
| 2622 |  | 1 | 79 | 3.5 | 0 | 3 | PVD only | RNAseq |
| 2683 |  | 1 | 39.5 | 15.5 | 1 | 3 | PVD only | RNAseq |
| 2520 |  | 2 | 37.5 | 2 | 1 | 2 | PVD only | RNAseq |
| 1347 |  | 8 | 68.5 | 12 | 1 | 3 | PVD only | qPCR |
| 1573 |  | 7 | 83 | 4.5 | 0 | 3 | PVD only | qPCR |
| 1676 |  | 6 | 65 | 4.5 | 0 | 3 | PVD only | qPCR |
| 2426 |  | 2 | 48.5 | 0 | 0 | 3 | PVD only | qPCR |
| 2567 |  | 1 | 87.5 | 7 | 0 | 3 | PVD only | qPCR |
| 2604 |  | 1 | 57 | 6 | 0 | 3 | PVD only | qPCR |
| 2024 |  | 4 | 41.5 | 8.5 | 0 | 3 | PVD only | qPCR |
| 1371 |  | 8 | 21 | 16 | 1 | 3 | PVD only | qPCR |
| 1854 |  | 5 | 42.5 | 18 | 2 | 2 | PVD only | qPCR |

**Supplementary Table S4.** Oligonucleotide primer sequences used for qPCR validation

| Gene symbol | Ensembl ID | Forward primer (5’-3’) | Reverse primer (5’-3’) |
| --- | --- | --- | --- |
| *GUSB* | ENSBTAG00000000704 | ACCATCGCCATCAACAACAC | TCCCGCGTAGTTGAAGAAGT |
| *SDHA* | ENSBTAG00000046019 | AAGACGTTCGACAGGGGAAT | ACTCGTCAACCCTCTCCTTG |
| *HSP90AB1* | ENSBTAG00000000778 | GCATGAAGGAGACGCAGAAG | TCCTTGAGCTGCTGTACACA |
| *RPS15* | ENSBTAG00000019718 | GCGACATGATCATTCTACCCG | GGTAGTGGCCGATCATCTCA |
| *NLRP3* | ENSBTAG00000001273 | CTTTCTGGACTCTGACCGGG | TCTGTCTGACCCCGAGGAAT |
| *IL1A* | ENSBTAG00000010349 | GCTCGGTTCAGCAAAGAAGT | GAGGTGGTCAATTTCAGAACTGT |
| *IL1B* | ENSBTAG00000001321 | CCCTGCAGCTGGAGGAAGTA | CTTCGATTTGAGAAGTGCTGATGT |
| *CXCL8* | ENSBTAG00000019716 | CATTCCACACCTTTCCACCC | CCTTCTGCACCCACTTTTCC |
| *CCL20* | ENSBTAG00000021326 | CAGCAAGTCAGAAGCAAGCA | TTTGGATCTGCACACACAGC |
| *TNFA* | ENSBTAG00000025471 | CCATCAACAGCCCTCTGGTT | TCACACCGTTGGCCATGA |
| *IL17A* | ENSBTAG00000002150 | CGTTAACCGGAGCACAAACT | TCCCAGATCACAGAGGGGTA |
| *TLR2* | ENSBTAG00000008008 | GGTTTTAAGGCAGAATCGTTTG | AAGGCACTGGGTTAAACTGTGT |
| *TLR4* | ENSBTAG00000006240 | GCTGTTTGACCAGTCTGATTGC | GGGCTGAAGTAACAACAAGAGGAA |
| *S100A8* | ENSBTAG00000012640 | ATTTTGGGGAGACCTGGTGGG | TGAACCAAGTGTCCGCATCC |
| *TGFB2* | ENSBTAG00000005359 | ACCCTCGGAAAATGCCATCC | CTGAACTCGGCCTTCACCAA |
| *IL10* | ENSBTAG00000006685 | GAAGGACCAACTGCACAGCTT | AAAACTGGATCATTTCCGACAAG |


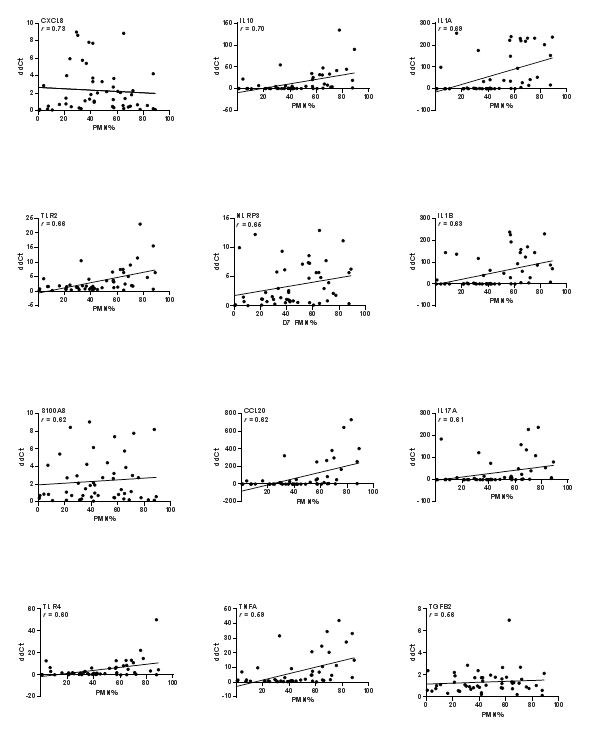


**Supplementary Figure S1.** Endometrial gene expression correlated with PMN% 7 days postpartum. Messenger (m)RNA expression of 12 genes was measured by qPCR (n=51) and ddCT values were plotted against the corresponding endometrial PMN% 7 days postpartum (DPP) as determined by cytological analysis. Correlation (*r^2^*) values are indicated for each gene.

**Supplementary Figure S2.** Epithelial and stromal gene expression markers differed between healthy cows without physiological inflammation at 7 DPP (low inflammation) and those with >18 % PMN at 7 DPP (high inflammation). Data is presented as read transcripts per million (TPM) from RNA-seq dataset (full dataset shown in Supplementary file 3).
